# Extrachromosomal Circular DNAs: Origin, formation and emerging function in Cancer

**DOI:** 10.7150/ijbs.54614

**Published:** 2021-03-02

**Authors:** Man Wang, Xinzhe Chen, Fei Yu, Han Ding, Yuan Zhang, Kun Wang

**Affiliations:** Institute for Translational Medicine, The Affiliated Hospital of Qingdao University, College of Medicine, Qingdao University, Qingdao 266021, China.

**Keywords:** extrachromosomal circular DNAs, gene amplification, drug resistance, tumor heterogeneity, cancer pathogenesis

## Abstract

The majority of cellular DNAs in eukaryotes are organized into linear chromosomes. In addition to chromosome DNAs, genes also reside on extrachromosomal elements. The extrachromosomal DNAs are commonly found to be circular, and they are referred to as extrachromosomal circular DNAs (eccDNAs). Recent technological advances have enriched our knowledge of eccDNA biology. There is currently increasing concern about the connection between eccDNA and cancer. Gene amplification on eccDNAs is prevalent in cancer. Moreover, eccDNAs commonly harbor oncogenes or drug resistance genes, hence providing a growth or survival advantage to cancer cells. eccDNAs play an important role in tumor heterogeneity and evolution, facilitating tumor adaptation to challenging circumstances. In addition, eccDNAs have recently been identified as cell-free DNAs in circulating system. The altered level of eccDNAs is observed in cancer patients relative to healthy controls. Particularly, eccDNAs are associated with cancer progression and poor outcomes. Thus, eccDNAs could be useful as novel biomarkers for the diagnosis and prognosis of cancer. In this review, we summarize current knowledge regarding the formation, characteristics and biological importance of eccDNAs, with a focus on the molecular mechanisms associated with their roles in cancer progression. We also discuss their potential applications in the detection and treatment of cancer. A better understanding of the functional role of eccDNAs in cancer would facilitate the comprehensive analysis of molecular mechanisms involved in cancer pathogenesis.

## Introduction

Extrachromosomal circular DNAs (eccDNAs), a major form of extrachromosomal DNAs, are widely present in various eukaryotic species, including drosophila 1, yeast 2 and humans 3. eccDNAs can be found in both normal cells and cancer cells 4. The biological roles of eccDNAs have attracted considerable attention, due to their heterogeneous origins and prevalence among almost all eukaryotes. eccDNAs may reflect genomic instability and plasticity 5. eccDNAs generally undergo unequal segregation during mitosis, driving and maintaining intercellular genetic heterogeneity of eccDNAs amounts 6. They also carry epigenetic modifications (e.g., transcriptionally active chromatin), fostering cellular fitness under positive selection pressures 7. More importantly, increasing evidence has proven the association between eccDNAs and cancer progression. eccDNAs are considered as the cytogenetic hallmarks of gene amplification in cancer 8. They act as a vehicle for the amplification of oncogenes and drug resistance genes 9, 10. The non-Mendelian inheritance pattern of eccDNAs is associated with a favorable transcriptional profile to force the expression of their constituent genes. Accordingly, eccDNAs serve as a pivotal player in tumor heterogeneity, evolution, aggressiveness and chemoresistance. eccDNA-driven intratumoral heterogeneity provides the foundation for which cancer cells can rapidly adjust to treatment and environmental pressures 11. It has been proposed that eccDNAs may be new therapeutic targets for cancer treatment. Moreover, eccDNAs can be identified in the bodily fluids of cancer patients and have the potential to be exploited as novel biomarkers for cancer 4. Here, we briefly summarize the origin, biogenesis and function of eccDNAs, and review the recent developments in deciphering the relationship between eccDNAs and cancer pathogenesis. In addition, we discuss their potential utility as a new type of biomarkers for cancer diagnosis, treatment evaluation and outcome prediction. The growing evidence from eccDNA study will likely provide insights for understanding the complexity of cancer biology and open up new avenues for the advancement of cancer intervention in clinical practice.

## Classification and origin of eccDNAs

The genome of eukaryotes consists of chromosomal DNA and extrachromosomal DNA elements which are physically excised from the chromosomes 3. The term “eccDNA” is now used to describe the full spectrum of circular DNAs in eukaryotes. eccDNAs are widely spread across various eukaryotes from yeast to human 12-14. eccDNAs can be divided into organelle eccDNAs such as mitochondrial DNAs (mtDNAs), and more flexible non-organelle eccDNAs such as double minutes (DMs), episomes, small polydispersed circular DNAs (spcDNAs) and microDNAs (Table 1) 15. Their sizes range from hundreds of base pairs (bp) to as large as several mega bases (Mb). Owing to universal existence and heterogeneous derivation, eccDNAs are considered to reflect genomic plasticity and instability.

Increasing evidence indicates that eccDNAs in eukaryotic cells usually carry interspersed repeat sequences or tandemly repeated genomic sequences 16-18. It can be concluded that tandemly repetitive DNAs are particularly prone to eccDNA formation 17. On the other hand, eccDNAs also derive from nonrepetitive DNA. Loon et al. 19 found that eccDNAs from HeLa S3 cells consisted of nonrepetitive or low-copy DNA sequences. Plus, some nonrepetitive spcDNAs were reported to be bordered on both sides by direct repeats of a mean length of 9-11 bp 20, 21. eccDNAs can originate from both coding and noncoding regions. For instance, oncogenes and drug resistance genes have been identified as predominant components of DMs 22. In addition, microDNAs are preferentially stemmed from exons, 5' untranslational regions (5' UTR) and CpG islands 23.

## The history of eccDNA discovery in eukaryotes

Hotta et al. 24 first identified DNA circles of diverse sizes within a preparation of mammalian DNAs. Contemporaneously, Cox et al. found small paired chromatin bodies in human cancer cells 25. These bodies were referred to as DMs since they are commonly identified in pairs. Indeed, DMs only constituted a small proportion (~30%) of the extrachromosomal particles of DNA in cancer cells 26. Like episomes, DMs are considered as circular vehicles of extrachromosomal gene amplification. A heterogeneous group of circular DNA species, ranging in size from 0.2 to 3.5 μm, was first observed in HeLa cell extracts by electron microscope 27. These eccDNAs were referred to as spcDNAs 28. spcDNAs ranged from several hundred bp to a few kilobases (kb) in length and predominantly originated from repetitive chromosomal sequences 29. The amount of spcDNAs was very low in normal cells 30. However, coordinated production of spcDNAs has been described in physiological processes, such as development and aging 31, 32. Increased biogenesis of spcDNAs in genetically unstable cells/tissues, such as cells exposed with carcinogens and tumor cells, has been documented, suggesting that they are tightly associated with genomic instability 33, 34. microDNAs are a newly characterized type of eccDNAs and arise from genic regions. Shibata et al. 14 identified a tiny circular DNA entity, derived from excised chromosomal regions, in mammalian cells. These eccDNAs were uniquely mapped to nonrepetitive sequences and were much smaller in length (~200-400 bp) than previously identified circular DNAs. Due to their small size and different origins, this family of eccDNA was named as microDNA. microDNAs exhibited lineage-specific or cell type-specific patterns in human cells 23, 35. The formation of microDNAs could produce a large pool of individual-specific copy-number variations of small fractions of the genome.

## Biogenesis of eccDNAs

**Figure 1:**
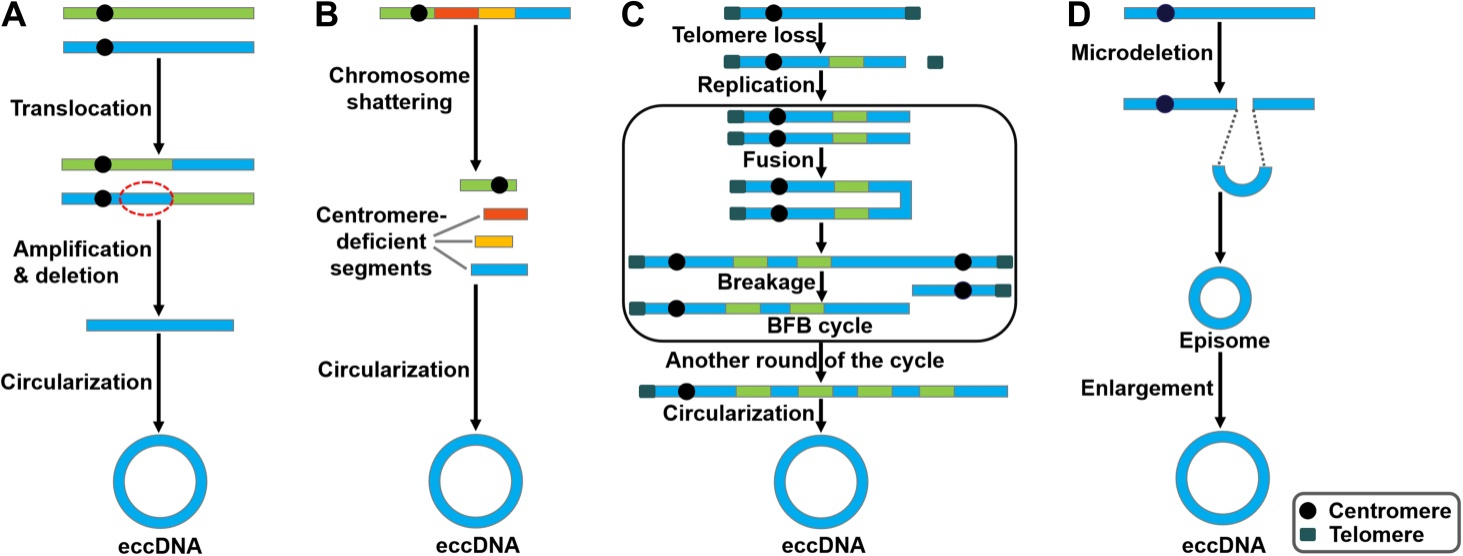
**Potential models of eccDNA biogenesis.** Four distinct models of eccDNA formation have been proposed.** (A)** The translocation-deletion-amplification model. Gene rearrangements take place near the translocation site on the chromosome. The fragment in proximity to the translocation breakpoints can be amplified, deleted and circularized, resulting in the genesis of eccDNAs.** (B)** The chromothripsis model. The shattering of the chromosomes can produce multiple acentric DNA segments. Some of these fragments can be self-ligated into circular DNA structures.** (C)** The breakage-fusion-bridge (BFB) model. The BFB cycle is initiated when a chromosome loses a telomere. The duplication of the chromosome during prophase results in the formation of two chromatids. The broken ends of the chromatids then undergo fusion, resulting in the production of a dicentric chromosome. Because of the presence of two centromeres, the fused chromatids form a bridge during anaphase that disrupts when the two centromeres are pulled to opposite poles. The segregation of each centromere into daughter cells leads to chromosome breakage and uneven distribution of genetic material. Specifically, one daughter cell gets a chromosome with inverted repetitive DNA sequences on its terminal, while the other gets a chromosome with a terminal deletion. Following DNA replication in the next cell cycle, the sister chromatids fuse once again and the BFB cycle can be repeated. These events lead to the amplification of DNA sequences residing near the telomere that eventually loop out and thus form extrachromosomal DNA elements.** (D)** The episome model. Episomes are derived from excision of small circular DNA. They can enlarge to form eccDNAs by over-replication or recombination.

There are several studies indicating contradictory results on the involvement of DNA replication process in eccDNA genesis. In mouse melanoma cells, eccDNAs were preferentially derived from major satellite DNA (MSD) 36. eccDNA production was increased in proliferating cells, implying that processes related to DNA replication participated in their generation. Accordingly, knockdown of DNA ligase IV (DNL4) could reduce eccDNA production. Nonhomologous end joining (NHEJ) was involved in eccDNA formation in mouse melanoma cells. Paradoxically, eccDNAs of telomere repeats (tel-eccDNAs) were produced *in vitro* using Xenopus egg extracts and sperm nuclei/naked DNA carrying telomere repeats 37. Aphidicolin, a specific inhibitor of DNA polymerase α, did not block the formation of tel-eccDNAs. The generation of tel-eccDNAs might be mediated by intrachromosomal homologous recombination between tandem telomere repeats. Likewise, Cohen et al. 38 revealed that eccDNAs were formed through excision of chromosomal sequences and did not require DNA replication by constructing a mammalian cell-free system. Moreover, they found that the process of eccDNA formation *in vitro* was energy-independent and required residual amount of Mg^2+^. Altogether, these results suggest that eccDNAs could be produced from the chromosomes mediated by recombination-dependent and -independent mechanisms. Loss- and gain-of-functional analyses in appropriate cell/animal models may be conducive to verifying the importance of DNA replication in the process of eccDNA biogenesis. The detailed process of eccDNA generation is still elusive. So far, four potential models for eccDNA formation have been proposed, including the translocation-deletion-amplification model, the chromothripsis model, the breakage-fusion-bridge (BFB) model and the episome model (Figure 1).

### The translocation-deletion-amplification model

In the translocation-deletion-amplification model (Figure 1A), translocation and amplification events cooperate to cause eccDNA formation 39. Specifically, gene rearrangements occur in close proximity to the translocation site 40. The segments adjacent to the translocation breakpoints are excised from their original chromosomal location and subsequently amplified, resulting in the formation of eccDNAs. It was found that the co-amplification of oncogenes *MYC* and AT motif binding factor 1 (*ATBF1*) was achieved through this model 41.

### The chromothripsis model

Chromothripsis is commonly found in cancer cells 42-44. Chromothripsis involves a catastrophic shattering process in specific genomic regions, producing a large number of sequence fragments (Figure 1B) 45. The generated segments are randomly stitched back together through the DNA repair machinery, resulting in locally clustered genomic rearrangement 46. In some cases, the genomic fragments can be circularized to form eccDNAs 47. Consequently, these fragments may be protected from degradation by eccDNA biogenesis. In small-cell lung carcinoma (SCLC), *MYC* was reported to be amplified via the chromothripsis model 48.

### The breakage-fusion-bridge (BFB) model

The BFB cycle proposed by McClintock is a well-established mechanism of genomic instability 49. BFB is characterized by the ligation and separation of sister chromatids. The BFB cycle begins with a telomeric loss on a chromosome (Figure 1C). During cell division the telomere-deficient chromosome replicates, and the broken ends of its two sister chromatids fuse together. This fusion generates a dicentric chromosome and an anaphase bridge 50. Because of the existence of two centromeres, the products caused by the breakage of the bridge are unequally partitioned into daughter cells, one with duplication and the other with deletion. The process of the BFB cycle can be repeated during various rounds of cell division. This leads to patterns of copy number increments of chromosomal fragments as well as fold-back inversions where duplicated fragments are arranged head-to-head 51. Finally, the amplified DNA circularizes to form eccDNAs via a recombinant mechanism 52.

### The episome model

Carroll et al. 53 revealed that small circular extrachromosomal molecules, referred to as episomes, took part in gene amplification. The episomes are generated via a recombination event that excises segments carrying a replication origin and its nearby genes (Figure 1D). They are considered as autonomously replicating submicroscopic precursors of eccDNAs. The enlargement of episomes leads to the formation of eccDNAs 54. The episome model was shown to mediate the biogenesis of *MYC*-containing eccDNAs in leukemia, SCLC, and neuroblastoma cells 55, 56.

Additional mechanism has been proposed for eccDNA production. In lymphoblastoid cells exposed to chemotherapeutics, cell apoptosis could facilitate eccDNA production 57. It is possible that DNA fragmentation during cell apoptosis may contribute to eccDNA formation. There remain major gaps in our knowledge regarding the nature of eccDNA molecules and mechanisms responsible for their formation. Advanced techniques and standardized procedures for eccDNA identification and characterization are required. This includes improving current sequencing and purification protocols to separate circular amplicons. It is essential to develop new computational tools that can be exploited to precisely assemble and construct circular amplicons. A better understanding of eccDNA biology will be helpful for unveiling the regulatory networks involved in eccDNA formation. eccDNAs may be generated through diverse mechanisms, including those associated with the deficiency in DNA repair and the creation of chromosome instability. It is necessary to unravel the detailed mechanisms involved in the generation of chromosomal instability during the process of eccDNA biogenesis. The genuine roles of the DNA repair pathways in eccDNA formation are yet to be delineated. It is unknown which DNA replication or repair pathways and proteins are implicated in producing distinct types of eccDNA. Given the contradictory results, there is still a lack of consensus as to the involvement of DNA replication in eccDNA genesis. Replication-defective cell models could be utilized to examine the amount and diversity of eccDNAs. Considering that the apoptotic pathway may participate in eccDNA formation, both living cells and dead cells contain eccDNAs. Differences in eccDNA repertoire between living cells and cells undergoing programmed death should be characterized, which will shed light on the biological function of eccDNAs. Moreover, additional studies must be carried out to delve into whether other cellular processes can facilitate the excision of DNAs from the genome. Importantly, appropriate cell models should be developed to explore the exact process of eccDNA formation. Additionally, the shuttling and translocation of eccDNAs may be highly regulated. The impact of eccDNA reintegration on genomic remodeling awaits thorough exploration. The internal and external factors that affect the copy number of generated eccDNAs remain to be identified.

## Functions of eccDNAs

In recent years, eccDNAs have become a focal point of scientific research, and increasing reports have showed their involvement in a wide range of biological processes. It seems that different types of eccDNA possess distinct functions. Understanding the biological function of eccDNAs will lead to elucidating epigenetic mechanisms behind gene regulation under normal and pathological circumstances.

### mtDNAs regulate innate immunity

**Figure 2:**
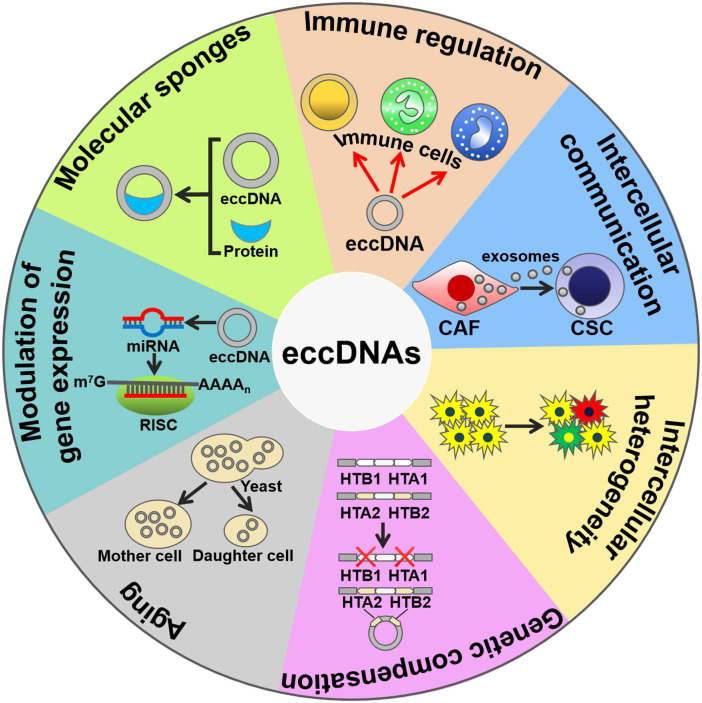
** The biological functions of eccDNAs.** eccDNAs play an important role in activating immune responses. eccDNAs mediate cell-to-cell communication and intercellular heterogeneity. eccDNAs facilitate genetic compensation and are associated with aging. Moreover, eccDNAs can be transcribed to produce noncoding RNAs, thus coordinating gene expression. Intriguingly, eccDNAs serve as sponges for transcription factors to indirectly modulate gene expression. CAF, cancer-associated fibroblast; CSC, cancer stem cell; RISC, RNA-induced silencing complex.

mtDNAs play a crucial role in triggering innate immunity (Figure 2). Collin et al. 58 revealed that purified mtDNAs induced inflammation and arthritis in mice, and that addition of mtDNAs to murine splenocytes enhanced the secretion of tumor necrosis factor-α (TNF-α). These findings suggest that mtDNAs function to initiating pro-inflammatory responses. Moreover, mtDNA is an activator of Toll-like receptor 9 (TLR9). CpG motifs within mtDNA could stimulate TLR9 signaling to activate p38 mitogen-activated protein kinase (MAPK) and neutrophil chemotaxis 59, 60. Accordingly, mtDNAs played a role in the TLR9-dependent inflammatory pathology of various diseases, such as acute liver injury and hypertension 61, 62. During cell apoptosis, B-cell lymphoma-2 (Bcl-2) homologous antagonist/killer (Bak)- and Bcl-2-associated X protein (Bax)-mediated mitochondrial damage caused the leaking of mtDNAs into the cytoplasm 63, 64. The released mtDNAs were recognized by the cyclic GMP-AMP synthase (cGAS)/stimulator of interferon genes (STING)-dependent DNA sensing pathway, thus leading to interferon-β (IFN-β) production and secretion by caspase-knockout cells.

### mtDNAs mediate cell-to-cell communications

Interestingly, mtDNAs can be encapsulated into extracellular vesicles (EVs) 65, 66. EVs are able to transfer mtDNAs between cells. For instance, cancer-associated fibroblast (CAF)-derived EVs transported mtDNAs to dormant breast cancer stem cells (CSCs) 67. The transferred mtDNAs promoted an exit from dormancy of CSCs and contributed to endocrine therapy resistance in breast cancer. Therefore, mtDNAs mediate the intercellular communication by functioning as important signaling molecules. Currently, only mtDNAs are found to be present in EVs. It is possible that other types of eccDNAs could be enclosed into EVs. Additional work is needed to verify this assumption. Moreover, the mechanisms underlying the sorting of mtDNAs into EVs are worthy of further investigation. EV-mediated shuttling of mtDNAs may facilitate the diffusion of diverse pathologies. Increasing knowledge of EV-derived mtDNAs will be conducive to thoroughly disclosing the function of mtDNAs and identifying novel pathological mechanisms of diseases.

### eccDNAs contribute to intercellular genetic heterogeneity

The eccDNAs can lead to deletions, mutations, replications, amplifications or translocation of genes, leading to intercellular genetic heterogeneity and adaptive evolution. Certain loci, including *DAZ4*, *HLA* and *KIR*, in human somatic tissue were prone to form circular products leaving chromosomal deletions 3. In a previous study, 1,756 eccDNAs were identified, and they covered 23% of the total yeast genomic information 2. Owing to their abundance, eccDNAs are one of factors associated with mutational and evolutionary characteristics of the eukaryotic genome. Selective pressure leads to a high requirement for the production of a specific gene, thereby causing the amplification of this gene locus. In *Saccharomyces cerevisiae*, the frequency of GAP1 eccDNAs and deletion of chromosomal *GAP1* were increased under nitrogen-limiting conditions 68. The formation of GAP1 eccDNAs in turn contributed to adaptation of *S. cerevisiae* to nitrogen-limited environments. During the adaptive evolution in *S. cerevisiae*, xylose isomerase (*XylA*) gene amplification occurred through genesis of self-replicating eccDNAs 21. The generated eccDNAs then integrated into their original locus on the genome, generating increasing numbers of tandem repeats within chromosomes. Higher gene dosage could provide a selective advantage for the proliferation or survival of *S. cerevisiae*. eccDNA formation followed by tandem gene amplification serves as a rapid means of adaptation to selective pressure during evolution. eccDNAs mediate gene translocation by integrating elsewhere in the genome. In cattle, a 492 kb chromosome 6 segment, *BTA6*, was circulated, reopened and integrated in the *BTA29* on chromosome 29 69. A circular shuttling DNA, encompassing a *BTA29* fragment, was translocated to the wild-type KIT locus within *BTA6* by homologous recombination. These serial translocation events finally led to color sidedness in cattle.

In *S. cerevisiae*, two gene pairs, *HTA1-HTB1* and *HTA2-HTB2*, encoded the histones H2A and H2B 70. *HTA2-HTB2* amplified via formation of eccDNAs under the condition in which *HTA1-HTB1* was deleted. The eccDNAs, which carried *HTA2-HTB2*, the histone H3-H4 locus, a centromere and the origin of replication, were generated through recombination between two Ty1 retrotransposon elements that flanked this region. Therefore, eccDNAs serve a critical role in compensating for genetic loss.

### eccDNAs are linked with aging in yeast

eccDNAs have been associated with aging. The aging yeast was enriched for high copy eccDNAs 71. The accumulation of eccDNAs during aging represents a genetic mechanism for conferring adaptive phenotypes, providing a beneficial outcome of aging in yeast. On the other hand, gathering of eccDNAs constituted a general cause of aging in yeast 71-73. eccDNAs harboring ribosomal RNA (rRNA) genes accumulated in aging yeast cells. These eccDNAs excised from the rDNA locus and replicated via their autonomously replicating sequence (ARS). eccDNAs were preferentially segregated to aging mother cells, thus limiting their copy number in daughter cells. Highly asymmetrical segregation of eccDNAs accounted for the restoration of youth in daughter cells. Since eccDNAs have been found in human normal cells and tissues, it is intriguing whether their accumulation could cause aging in higher eukaryotes.

### eccDNAs can be transcribed into noncoding RNAs

Interestingly, eccDNAs can be transcribed within cells, suggesting that they may have functional roles in cell physiology. Greenwood et al. 74 revealed that transcription occurred multiple times around extrachromosomal circular rDNAs without termination in *Euglena gracilis*. microDNAs harboring microRNA (miRNA) coding sequences were transcribed to form functional miRNAs that silenced their endogenous mRNA targets 75. Moreover, microDNAs derived from exons regulated the expression of their host genes by producing small interfering RNAs (siRNAs). Therefore, microDNAs can coordinate gene expression through the RNA interference pathway. In *Oxytricha*, abundant eccDNA molecules from nonrepetitive micronucleus (MIC)-limited sequences were generated during genome rearrangement 76. These eccDNAs served as templates for the transcription of long noncoding RNAs (lncRNAs). Further studies are needed to investigate the function of the lncRNAs produced from eccDNAs in *Oxytricha*. The exact mechanisms underlying the transcription of microDNAs remain to be clarified. In addition, microDNAs acted as protein sponges to combine with RNA polymerase subunits 75. The mechanisms behind the sponging function of microDNAs await further exploration. Specific motifs or structural characteristics of microDNAs may be critical for protein sequestration. Additional work is necessary to identify the essential elements within microDNA. It is still unclear whether microDNAs can sequester noncoding RNAs (ncRNAs), such as miRNAs and lncRNAs. Much effort needs to be devoted to studying the interaction between microDNAs and ncRNAs.

### Telomeric circles are involved in restoring telomere length

Telomeric circles (t-circles) function as pivotal participants in telomere maintenance. In cancer cells, increased telomerase activity caused extended telomere length 77. Cancer cells limited the telomere length by trimming telomeric DNA from the chromosome ends, possibly by the generation and release of t-circles. Meanwhile, cancer cells prevented the shortening of their telomeres through a recombination-mediated alternative lengthening of telomere (ALT) mechanism 78. ALT-positive cells commonly have telomeres that are highly heterogeneous in length. ALT cells harbored unique nuclear structures called ALT-associated promyelocytic leukemia (PML) bodies (APBs), which contained telomeric DNA and telomere-associated proteins 79. DNA damage increased the number of APB-positive ALT cells and led to an ALT-specific induction of circular extrachromosomal telomeric repeat (ECTR) DNA 80. Circular ECTR originated from double-strand breaks (DSBs) that cleaved off t-loops or from recombination events that caused the conversion of t-loops into free circles. t-circles with a nick or gap served as a template for rolling circle replication (RCR) 81. Strand invasion from the telomeric 3' overhang induced the start of RCR on a t-circle template. The newly generated telomeric DNA molecules were processed into smaller linear telomeric DNAs, each of which was circularized to act as an RCR template. Therefore, the roll-and-spread mechanism facilitated constant propagation of t-circles in ALT cells. Additionally, t-circle production is not influenced by the expression of telomerase in ALT-positive fibroblast cells. C-rich circles (C-circles) are single-stranded extrachromosomal telomeric circles. It was hypothesized that C-circles were generated from t-circles 82. The telomere end structure was tightly linked with the formation of C-circles 83. Specifically, ALT cells contained C-rich single-stranded overhangs. The telomere length in ALT cells gave rise to a lack of sufficient telomere-binding proteins, thereby causing the alteration in telomeric chromatin. Telomeres acted as substrates for C-circle formation. Additionally, telomerase could inhibit the generation of C-strand overhangs and C-circles 83.

### eccDNAs show promise as circulating biomarkers

Increasing evidence has proven the existence of eccDNAs in circulation system 84, 85. microDNAs were detectable in serum and plasma samples from cancer patients 4. More importantly, circulating microDNAs were longer in patients carrying tumors than those in the same patients after surgical resection of the tumors. Circulating eccDNAs may represent prospective biomarkers for diagnosis and outcome prediction of diseases. At present, studies on cell-free eccDNAs are limited to plasma and serum samples. The presence of eccDNAs in other bodily fluids, such as saliva and urine, should be examined.

Taken together, eccDNAs play a role in eliciting innate immune responses, mediating cell-to-cell communication, advancing genetic heterogeneity, compensating for genetic loss, aging, regulating gene expression and molecular sponging (Figure 2). eccDNAs may be a new source of genetic materials for liquid biopsy. It should be noted that many gaps still exist in our understanding with regard to the biological functions of eccDNAs. Extensive work remains to be done in understanding the impact of eccDNAs on cell physiology. The influence of eccDNA excision and translocation on the structure and function of genes needs to be determined in future studies.

## Cancer-derived eccDNAs

eccDNAs have been discovered in cancer tissues/cells by high-throughput sequencing. Recently, over 18,000 eccDNAs were identified in a pan-cancer analysis of ATAC-seq libraries from 23 cancer types, including glioblastoma multiform (GBM), lung adenocarcinoma (LUAD) and stomach adenocarcinoma (STAD) 86. Notably, eccDNAs carrying epidermal growth factor receptor (*EGFR*) gene were identified in patient-derived GBM cell lines. Koche et al. 87 provided a comprehensive map of eccDNAs in neuroblastoma. They identified 5,673 eccDNAs, with an average size of 2,403 bp, per neuroblastoma. These eccDNAs were associated with somatic genomic rearrangements in neuroblastoma, leading to oncogenic remodeling with significant functional and clinical outcomes. On average 3,669 unique microDNA clusters were identified per human lymphoblastoid cell line (LCL) sample 57. These microDNAs were mainly derived from the active regions of the genome. The number and average length of microDNAs were influenced by chemotherapeutic treatment and cell sensitivity status. These observations suggested that chemotherapy-induced apoptosis partially regulated microDNA diversity in LCLs. Sen et al. 88 identified eccDNAs with a size ranging from 400 bp to 1 kb in the bone marrow cells of patients with acute leukemia by Alu-polymerase chain reaction. Particularly, microDNAs derived from chromosomal genomic sequences were released from normal and tumor tissues into the circulation and were often longer than linear cell-free DNAs 4. Moreover, microDNAs from human lung cancers were longer than that from the paired normal tissues. eccDNAs may reflect disease status and could be used as novel extracellular nucleic acid biomarkers for cancer. spcDNAs were discovered in lymphocytes of healthy volunteers and various cancer cell lines 89. The quantity and sequence composition differed between normal lymphocytes and cancer cells. The amount of spcDNAs in cancer cells was over 3-fold higher than in normal lymphocytes. Notably, spcDNAs from cancer cells harbored abundant repetitive sequences (Alu, LINE-1 and telomeric elements) compared with those from normal lymphocytes. Altogether, spcDNAs may be linked with genomic rearrangement and instability during carcinogenesis.

Increasing evidence has indicated that circular DNA molecules are important structures in oncogene amplification in cancer cells 90, 91. For instance, oncogenes *EGFR* and* c-MYC* were found to be more amplified in human cancer tissues than normal tissues 6. The copy number of the two oncogenes in eccDNA amplification even exceeded their copy number in chromosomal amplicons. A recent report indicated that the oncogenes (e.g. *EGFR* and *MYCN*) and their endogenous enhancers were maintained and amplified as extrachromosomal amplicons 7. The preservation of contacts to enhancers and topological changes driven by amplification and circularization affected the ability of oncogenes to regulate cell viability. Cells derived from gliomas contained extrachromosomal amplicons carrying amplified copies of the *EGFR* gene 92. All of the amplicons originated from a single precursor in each tumor. This eccDNA was generated via the simple joining of the ends of a chromosomal segment overlapping the *EGFR* gene through the microhomology-based NHEJ pathway. eccDNA molecules including amplified *MYC* genes were universally present in human cancer cells 93, 94. The *MYCN* gene was amplified as DMs in neuroblastoma and SCLC cell lines 56. The DMs were generated by excision from the chromosome and formation of circular intermediates. Heterogeneous *MYC*-carrying DMs coexisted within the same leukemia cell population of patients with acute myeloid leukemia (AML) 95. The episome model might be responsible for DM genesis 55. The amplicon bearing the *MYC* gene was generated by a postreplicative excision from its original chromosomal location. The circularization of this amplicon might involve error-prone NHEJ pathway. It was likely that the scattered acentric DMs evolved toward ring chromosomes stabilized by neocentromeres, thus providing a selective advantage to leukemia cells.

Compared with reports on ncRNAs, there are relatively few studies on eccDNAs. Therefore, it is necessary to comprehensively identify and characterize eccDNAs in cancer. Profiling the landscape of eccDNAs will provide important clues on the functional role of eccDNAs in carcinogenesis and cancer progression. Much more efforts should be made to compare the amount, sequence composition and topological characteristics of eccDNAs between cancer patients and normal controls. The screening of cancer-specific or -associated eccDNAs will lead to the discovery of novel biomarkers for cancer diagnostics and promising targets for anticancer therapeutics.

## Emerging functions of eccDNAs in cancer

### eccDNAs foster oncogene amplification and intratumoral heterogeneity

**Figure 3:**
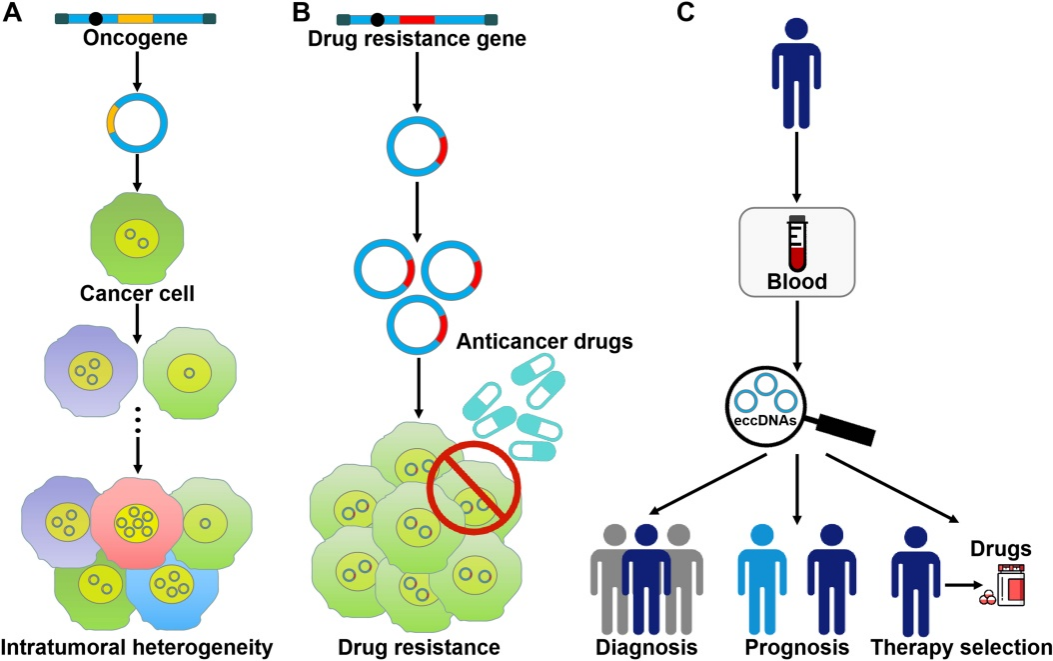
** The roles of eccDNAs in cancer pathogenesis. (A)** eccDNAs act as a vehicle for the amplification of oncogenes. Thus, they serve a function in tumor heterogeneity. **(B)** eccDNAs confer drug resistance to cancer cells by increasing the amplification of drug resistance genes.** (C)** The clinical values of eccDNAs in cancer. eccDNAs have been found in human blood samples. The aberrant expression of eccDNAs in the peripheral blood from cancer patients makes them ideal candidates for non-invasive biopsy biomarkers in cancer.

eccDNA formation is an important mechanism responsible for the amplification of oncogenes (Figure 3A). The massively elevated expression of oncogenes amplified on eccDNA is attributed to its increased DNA copy number with high transcription levels and enhanced chromatin accessibility 9. eccDNAs bearing amplified copies of oncogenes are unequally segregated during mitosis, leading to increased heterogeneity among offspring cells. The heterogeneity provides cancer cells with a pool of genomic alterations that enable them to adapt to the environment under diverse pressures. DMs are the cytogenetic hallmarks of genomic amplification in cancer. DM molecules are randomly generated in cancer cells 5. With increasing number of DNA copies, the amplified DNA region is prone to acquisition of an amplification-linked extrachromosomal mutation (ALEM). The extrachromosomal nature of ALEM contributes to the altered amounts of mutated oncogenes. Cells with the highest number of DMs carrying the driver ALEM would have a proliferative advantage. An in-depth analysis of eccDNAs in paired diagnosis and relapse tumors from GBM patients suggested that eccDNAs readily evolved and contributed to tumor heterogeneity 96. During the process of cancer pathogenesis, new eccDNAs could be formed at any time and every eccDNA acquired new somatic mutation, resulting in competition among distinct versions of eccDNAs. Some eccDNAs formed before or at the time of diagnosis were removed by treatment, while those eccDNAs that favored tumor growth and conferred drug resistance could survive and expand 96. Collectively, oncogene amplification on eccDNAs endows cancer cells with a selective growth advantage.

The coexistence of *cyclin D1* amplification in DMs and homogeneously staining regions (HSRs) was confirmed in primary bladder tumors 97. Patients with DM-carrying tumors had a remarkably shorter overall survival rate than patients without DMs in their primary tumors. The amplification of *cyclin D1* on DMs might be associated with clinical characteristics of patients. The oncogene *Sei-1* was capable of inducing DM formation 98. The oncogene *Met* carried on DMs was significantly amplified and overexpressed. The Met signaling cascade in turn promoted the *Sei-1*-induced DM generation. High copy number amplification of eukaryotic initiation factor 5A2 (*EIF5A2*) was detected in ovarian cancer (OC) cells in the form of DMs 99. *EIF5A2* overexpression was significantly correlated with the advanced stage of OC. Downregulation of *EIF5A2* by the reduction of DM copy numbers suppressed the growth of OC cells. Thus, EIF5A2 served a vital role in OC pathogenesis. Likewise, ribosomal L22-like1 (*RPL22L1*) was amplified via DMs in OC 100. RPL22L1 was tightly associated with stage, invasion and lymph node metastasis of OC patients. *In vitro* and *in vivo* evidence indicated that RPL22L1 could promote OC development. Gemcitabine was able to enhance the incorporation of DMs into micronuclei in OC cells 101. Moreover, the expression level of oncogenes *EIF5A2*, *MYCN* and myeloid cell leukemia-1 (*MCL-1*) present on DMs was decreased upon gemcitabine treatment. Accordingly, gemcitabine restrained the growth, colony formation and invasion of OC cells.

The universal occurrence of eccDNA-driven oncogene amplification was verified in glioblastoma 8. During cell culture and xenografting, eccDNAs exhibited unevenly distributed across offspring cells, contributing to the regulation of the oncogenic potential of cells. Thus, eccDNAs play an important role in enhancing genomic diversity during tumor evolution. The DM-carried genes (e.g., *NSMCE2*, *MYC* and *FGFR2*) were identified to be amplified and overexpressed in human colorectal cancer (CRC) cells 102. Inhibiting the expression of DM-carried genes could cause the loss of DMs, which in turn resulted in the reduced amplification of DM-carried genes. Depletion of DM-carried genes also caused the suppression of cell proliferation and invasion, suggesting that the DM-carried genes promoted the invasive feature of cancer cells. Oncogenes on eccDNAs may be vulnerable to loss, leading to a less aggressive biological behavior in cancer cells 103. In contrast, oncogenes located on the chromosome are hardly excluded. In human leukemia cells, *c-MYC* could shift from an eccDNA to an intrachromosomal site. It is likely that integration of the oncogene into the chromosomal locus facilitates tumor progression.

Genetic aberrations were discovered in the leukemia cells of a breast cancer patient who developed secondary AML after chemotherapy 104. Further study indicated the occurrence of fragmental losses and gains in chromosomes. It turned out that *MYC* and lysine-specific methyltransferase 2A (*KMT2A*) were concurrently amplified on different DMs. These alternations may contribute to the pathogenesis of secondary myeloid malignancies. DMs in myeloid neoplasms generally contained *MYC* or mixed lineage leukemia (*MLL*) gene amplification and were linked with myelodysplasia or therapy-related disease 105. These findings suggest that DMs are involved in carcinogenesis and cancer pathogenesis by acting as a vehicle for extrachromosomal oncogene amplification.

There have been a large number of reports showing oncogene amplification in the form of DMs in cancer. Cancer cells harboring increased oncogene expression and copy number acquire a competitive advantage and possess the ability to rapidly fit changing environment. DMs play an important role in tumor heterogeneity, evolution and development. The mechanisms that drive oncogene amplification within DMs are worthy of further investigation. The critical factors that regulate the formation and amount of oncogene-carried DMs need to be determined. Furthermore, it is unknown whether the high-level amplification of oncogenes occurs within other kinds of eccDNAs. Thus, further research focusing on the oncogene amplification via other types of eccDNAs is required.

### eccDNAs contribute to drug resistance in cancer cells

eccDNAs predominantly bear extrachromosomal amplification of drug resistance genes (Figure 3B). The acquirement of drug resistance in cancer cells is associated with drug resistance gene amplification on eccDNAs. In tumor cells from a SCLC patient who received methotrexate (MTX), a large quantity of DMs was discovered, and the gene coding for dihydrofolate reductase (DHFR) was amplified and overexpressed 106. During serial passage of this cell line in drug-free medium, the number of DMs and the expression level of DHFR were declined. As a result, cancer cells showed increased MTX sensitivity. These findings suggested that DMs were associated with the development of drug resistance. Reportedly, homologous recombination activity was increased in DM-carrying MTX-resistant colon cancer cells when compared to MTX-sensitive cells 107. Homologous recombination might participate in DM genesis and the suppression of DM exclusion by fostering cell cycle arrest at the G2/M checkpoint. Importantly, inhibition of homologous recombination activity reduced the copy number of DM-form amplified genes, including *DHFR*, zinc finger FYVE-type containing 16 (*ZFYVE16*) and mutS homolog 3 (*MSH3*), and enhanced drug sensitivity in MTX-resistant cells. Similarly, suppression of NHEJ blocked DM generation, inhibited MTX resistance and cell proliferation in MTX-resistant colon cancer cells 108. Therefore, the DSB repair pathway may represent a novel target to improve therapeutic outcome by limiting extrachromosomal amplification in cancer.

EGFRvIII originates from a genomic deletion of the *EGFR* gene and predominantly maintains on DMs 92, 109. In GBM, EGFR is commonly mutated, consequently giving rise to the oncogenic variant EGFRvIII 110. Dynamic modulation of EGFRvIII expression by DM mediated resistance to EGFR inhibition in GBM 10. GBM cells could acquire resistance to erlotinib, an EGFR-targeted drug, by eliminating mutant EGFR from DMs. In the absence of erlotinib, the mutant EGFR gene reemerged on DMs, re-sensitizing GBM cells to erlotinib-induced cell death. Collectively, cancer cells are able to evade drug therapy that targets oncogenes resided on eccDNAs by the “hide and seek” mechanism. Partial depletion of mtDNAs induced aerobic glycolysis in CRC cells and decreased adenosine triphosphate (ATP) production 111. mtDNA content reduction contributed to reversible resistance to 5-fluorouracil- and oxaliplatin-induced apoptosis. mtDNA reduction might help cancer cells to gain a survival advantage.

In human epidermoid carcinoma cells, the amplified multidrug resistance 1 (MDR1) genes were contained in DM molecules 112. The upregulation of MDR1 caused resistance to various anticancer drugs. The MDR amplification event was analyzed in colchicine-treated human epidermoid cancer cells 113. During the colchicine selection, circular DNAs (890 kb) harboring MDR dimerized to large DM structures (1,780 kb) by intramolecular homologous recombination, which then dimerized to form the larger DMs (3,560 kb). Dimerization of circular amplicons served as a crucial mechanism for DM generation and MDR gene amplification. Colchicine exposure also induced the mutation of MDR gene. The mutated MDR gene residing on the DM structures conferred enhanced drug resistance to cancer cells. In addition, fractionated ionizing radiation reduced the extrachromosomal copy number of MDR1 in human epidermoid cancer cells by inducing the entrapment of eccDNAs carrying MDR1 in micronuclei 114. As a result, the expression level of P-glycoprotein was decreased and multidrug resistance of cancer cells was attenuated. Therefore, radiation-induced loss of extrachromosomally amplified MDR1 gene might help to improve the efficacy of anticancer therapies.

Extrachromosomal amplification of drug resistance genes serve as a vital mechanism responsible for cancer drug resistance. eccDNAs carrying drug resistance genes can drive intratumoral heterogeneity and contribute to extreme copy number amplification through their uneven segregation. Cancer cell-derived eccDNAs increase the diversity of genetic differences among offspring cells, thus conferring a proliferative or survival advantage to cancer cells under drug pressure. Suppression of eccDNA biogenesis may improve the therapeutic efficacy of anticancer drugs. More studies are needed to investigate eccDNA-mediated drug resistance in cancer, which will provide new insights into the epigenetic mechanism underlying cancer pathogenesis.

### eccDNAs may be used as promising biomarkers for cancer

mtDNA copy number variation has been correlated with cancer 115. High mtDNA copy number in peripheral blood leukocytes (PBLs) was associated with increased risk of prostate cancer (PCa) and high tumor burden in PCa patients 116. Therefore, quantification of mtDNA content in PBLs might be helpful to diagnosis of PCa and evaluation of tumor burden. Paradoxically, another report indicated that low mtDNA copy number in PBLs correlated with aggressive PCa at diagnosis and might be useful in predicting poor prognosis of localized PCa patients 117. The potential of mtDNAs as a PCa biomarker needs to be investigated in future studies. Levels of mtDNA copy number in peripheral blood of patients with breast cancer were markedly higher than in that of healthy controls 118. Elevated levels of mtDNA copy numbers were correlated with an increased risk of breast cancer. Circulating cell free mtDNAs were markedly increased in patients with breast cancer and benign breast lesions 119. Furthermore, circulating mtDNAs could distinguish patients with breast cancer from healthy controls with good sensitivity. Circulating mtDNAs may be exploited as a new biomarker for the diagnosis of breast cancer. The copy number of exosomal mtDNAs was significantly increased in patients with serous epithelial ovarian cancer (SEOC) compared to healthy controls 120. The mtDNA copy numbers might be a signal of cancer development. Exosomal mtDNAs may hold promise as new cancer biomarkers. Further studies are needed to assess the clinical utility of exosomal mtDNAs in cancer detection and prognosis. A meta-analysis showed that the elevated amount of mtDNAs was significantly correlated with the risk of lymphoma, breast cancer and melanoma, while it was negatively linked with hepatic carcinoma 121. mtDNA copy number was lower in PBLs of CRC patients than healthy controls 122. Low mtDNA copy number was markedly correlated with an increased risk of CRC. These findings demonstrated that mtDNA copy number might represent a long-term biomarker in predicting the risk and prognosis of CRC. Similarly, the mtDNA copy number was remarkably lower in PBLs of patients with endometrial cancer than normal controls 123. Low mtDNA content was significantly associated with increased risk of endometrial cancer. Although the relationship between mtDNA amount and cancer risk appears to be coordinated by various genetic and cellular factors, the exact mechanism is still obscure and remains to be further elucidated. As is known, increased mtDNA content is markedly linked to varied oxidative stress, aging, immune activation, and response to environmental exposure. Extensive studies are required to assess crucial factors that affect mtDNA copy number and to provide clues on the molecular mechanisms of mtDNA copy number alternation in cancer progression.

Reportedly, cancer patients had higher plasma concentrations of mtDNA compared with healthy controls 124. The plasma concentrations of mtDNA were decreased during radiation therapy in most of cancer patients. Circulating mtDNA levels could be useful in monitoring the response to radiation therapy in cancer patients. However, further studies involving a large patient cohort are demanded to completely assess the clinical value of circulating mtDNAs. Patients with pediatric acute lymphoblastic leukemia (ALL) had higher mtDNA content compared with healthy controls 125. mtDNA copy numbers were obviously reduced in patients following chemotherapy. In addition, patients with high mtDNA copy numbers displayed inferior survival than those with low mtDNA copy numbers. High mtDNA copy number may represent a promising biomarker for predicting poor survival in ALL patients.

It was reported that high mtDNA copy number in peripheral blood of cancer patients predicted a poor cancer prognosis while increased mtDNA copy number in tumor tissues was remarkably correlated with better overall survival in cancer patients 126. However, the correlation between mtDNA content and cancer prognosis may vary depend on the type or stage of cancer. Low mtDNA copy number in CRC tissues correlated with poor prognosis in CRC patients and might reflect multiple malignant variations, probably involving cancer growth and invasiveness 127. By contrast, the mtDNA content was higher in colon cancer tissues than matched adjacent colon tissues 128. High mtDNA content in tumor tissues was associated with tumor growth, advanced tumor-node-metastasis (TNM) stage and poor prognosis in patients with colon cancer. The prognostic value of mtDNAs in breast cancer patients was also identified 129. Patients with low mtDNA amount exhibited a high metastatic potential and a significant unfavorable prognosis compared to patients with high mtDNA amount. The clinical relevance of mtDNA content determination in patients with breast cancer needs to be further verified. Low mtDNA copy number in breast cancer tissues could predict better outcome for patients receiving anthracycline-containing chemotherapy 130. It was probable that cancer cells with low mtDNA content were more susceptible to anthracycline therapy. mtDNA content alteration in breast cancers may provide helpful clues for therapeutic decision-making.

The amount and nature of eccDNAs in cancer cells differ from that of normal cells 131. The frequency of eccDNAs may vary by cancer type 6. Extracellular free eccDNAs can be detected in cancer tissues and blood samples of cancer patients 87, 132. Therefore, it has been postulated that eccDNAs, especially microDNAs, could be used as a novel type of biomarkers for cancer detection, treatment assessment and prognostic surveillance (Figure 3C). Nevertheless, there are still many challenges that need to be addressed before the clinical application of eccDNAs. It has been found that a large number of cell-free eccDNAs have unique sequences 85. Accordingly, substantial studies are required to characterize the hotspots of eccDNA origination in different cancers and to identify the targeted region within eccDNAs. Moreover, it remains unclear whether eccDNAs exist in other bodily fluids including saliva and urine. It is necessary to test the presence of eccDNAs in all bodily fluids. In addition, current studies mainly focus on the clinical value of mtDNAs. The potential utility of other types of eccDNAs as cancer biomarkers requires to be verified.

## Conclusions and perspectives

In eukaryotes, the cellular landscape has different genetic components, such as chromosomal nuclear DNAs, coding RNAs, ncRNAs and so on. Besides chromosomal nuclear DNAs, eccDNAs are universally present in the nucleus and cytoplasmic organelles (e.g. mitochondria) of normal and malignant cells. In the past decades, extensive efforts have been made to uncover the mechanisms of eccDNA biogenesis. eccDNA formation may involve DNA replication-dependent or -independent processes. eccDNAs can be derived from either repetitive or nonrepetitive chromosomal sequences. Although several models have been raised to describe the biogenesis of eccDNAs, the precise mechanism and direct proof for these models deserve further research. It is still unclear how the segments of linear chromosomes circularize. More efforts are needed to characterize the topological structure and sequence composition of eccDNAs. Little is known about the mechanisms leading to the variation of copy numbers of eccDNAs. Additional work is required to figure out whether eccDNAs have the ability to self-replicate. At present, there is no consensus standard for eccDNA analysis. Different approach used in eccDNA study may have a marked influence on analytical results. Thus, it is important to standardize the procedure of eccDNA enrichment and data analysis. Further research is necessary to detect the basis of eccDNA formation across diverse species. eccDNAs have been studied for their role in genomic plasticity, instability, evolution, mutation and carcinogenesis. However, the functional roles of eccDNAs are yet to be fully delineated. Multiple studies have focused on the gene amplification capabilities of large-sized DMs. However, the role of microDNAs in gene amplification remains unclear. The gene amplification capabilities of smaller eccDNAs should be confirmed. Furthermore, there is a lack of direct evidence supporting the contribution of smaller eccDNAs to chromosome instability. These unanswered questions warrant further investigation to strengthen our knowledge regarding eccDNA function. The turnover of eccDNAs in cells or bodily fluids needs to be studied, which will provide new insights into their functions. Since cells produce diverse eccDNAs with heterogeneous origins, the connection between eccDNAs and different physiological/pathological states of normal cells is an intriguing area of ongoing research. Existing evidence indicates that a fraction of eccDNAs reside in the nucleus and can be transcribed. eccDNAs are likely to act as active gene transcripts and gene expression modulators. Thus, it is essential to define the impact of these eccDNAs and their gene products on the phenotype of cells.

eccDNAs play a crucial role in cancer progression. eccDNAs confer an ability to amplify oncogenes and drug resistance genes. The proteins implicated in producing eccDNAs from the chromosome may serve as therapeutic targets for cancer. Therapies targeting eccDNAs may contribute to overcoming drug resistance in cancer. Nevertheless, the regulatory roles of eccDNAs in cancer pathogenesis remain to be verified. It is obscure whether eccDNAs directly regulate cancer cell proliferation, invasion and metastasis. Loss-of-functional analysis may be helpful to elucidate the effects of eccDNA depletion on cancer development. A comprehensive landscape of eccDNAs in neuroblastoma was previously profiled 87. eccDNAs played a vital role in cancer genome remodeling with important functional and clinical outcomes through chimeric circularization and reintegration into the genome. eccDNA-related genomic rearrangements may act as a novel mechanism responsible for cancer pathogenesis. However, a variety of eccDNAs have yet to be characterized. Circle-derived rearrangements have previously been underestimated in genome sequencing analysis. Therefore, an in-depth investigation into eccDNA-associated arrangements will expand our understanding of cancer genome remodeling and provide new insights into the epigenetic mechanisms behind cancer pathogenesis. Wu et al. 9 found the highly accessible chromatin of eccDNAs in cancer cells. It seemed that eccDNAs promoted cancer progression through increased transcription of their constituent oncogenes or alteration of their chromatin organization. The chromatin topology of eccDNAs can lead to positive selection and cellular adaptation to changing environment via accessibility to regulatory elements and transcription machinery. In comparison with their linear counterparts, eccDNAs display distinctive chromatin landscape. Thus, the chromatin structural features of eccDNAs are worthy of systematic exploration. Cancer-associated sequence characteristics of eccDNAs remain to be identified. The maintenance of the contact between genic sequences and regulatory elements form an epigenomic mechanism underlying cancer aggressiveness. A thorough understanding of eccDNA chromatin organization will shed lights on the effects of eccDNA structures on oncogene function, thus connecting eccDNA biology with cancer epigenetics.

Notably, circular DNAs hold tremendous promise as therapeutic ncRNA antagonists. Meng et al. 133 developed small circular single-stranded DNA-9 (CSSD-9). They found that CSSD-9 increased the expression of co-silenced tumor suppressor genes (*KLF17*, *CDH1* and *LASS2*) by decoying miR-9 that targeted these genes. The transfection of CSSD-9 via nanoparticles suppressed the proliferation and metastasis and favored the apoptosis of malignant cancer cells *in vitro* and *in vivo*. These results suggested that CSSD-9 might be used as therapeutic miRNA inhibitors to upregulate tumor suppressor genes and reduce tumor malignancy. Circular DNAs may be effective for sequestering miRNAs and provide a new perspective for the intervention of cancer. From a therapeutic viewpoint, an ideal ncRNA interference technology should display high stability and ncRNA inhibitory activity. eccDNAs may carry the favorable feature of resistance against nuclease degradation. Nevertheless, it remains to verify whether natural eccDNAs can act as ncRNA sponges. Specific sequences involved in the interaction between eccDNAs and ncRNAs are required to be identified. The feasibility of eccDNAs toward the regulation of tumor suppressor genes or oncogenes *in vitro* and *in vivo* models warrants further exploration. In addition, substantial studies should be carried out to evaluate the effectiveness and safety of eccDNA-based anticancer therapeutics. There are several difficulties that must be addressed going forward. One of the challenges is the risk of immune stimulation. The eccDNAs may be recognized by the innate immune system of target cells. Introduction of a large quantity of eccDNAs may induce non-specific interactions owing to the activation of innate immunity. It is important to design eccDNAs which can act at a low concentration. Additionally, the utilization of efficient delivery systems may ensure that eccDNAs can be applied at the lowest concentration. The off-target effect is another challenge with eccDNA-based therapies. The off-target effect can cause the suppression of ncRNAs that should not be targeted, which may lead to deleterious consequences. The screening and design of eccDNAs must be carefully performed to avoid this issue. eccDNA-based therapeutics that allow long-lasting and steady ncRNA-trapping at a low concentration may be feasible in enhancing therapeutic efficacy with a low risk of the off-target effect.

Increasing evidence has demonstrated the potential utility of eccDNAs as a source of novel biomarkers for cancer detection and prognosis. Since eccDNAs can be detected in peripheral blood from cancer patients, cell-free eccDNAs may represent minimally invasive biomarkers for cancer. Despite the benefits of eccDNAs as potential novel biomarkers, there are several obstacles to be overcome. First, there are still many undescribed eccDNAs in cancer cells. Additional studies should be performed to screen and identify eccDNA candidates. It is believed that more and more eccDNAs will be discovered and characterized due to recent technological developments such as super-resolution microscopic method and next-generation sequencing. The difference in eccDNA contents between cancer patients and healthy controls must be ascertained. Deregulated eccDNAs may be useful in distinguishing cancer patients from healthy controls. Secondly, the approaches for eccDNA isolation, enrichment and quantification should be defined to ensure the accuracy of analytical results. Thirdly, the potential of eccDNAs as effective biomarkers is worthy of intensive investigation. Although many studies have indicated the altered amount of eccDNAs in cancer, the diagnostic and prognostic values of eccDNAs are elusive. Therefore, more clinical studies are necessary to assess the diagnostic/prognostic performance of eccDNAs in cancer.

In conclusion, eccDNA study is still in its infancy, and the biological role of eccDNAs in cancer pathogenesis is just starting to be illuminated. There are many areas to address in this field, such as the biogenetic process of eccDNAs and the influence of eccDNAs on cell physiology. Increased research on the functional role of eccDNAs in cancer will broaden the horizons in the field of cancer biology and open up new opportunities for cancer diagnosis and therapy.

## Figures and Tables

**Table 1:** Classification of eccDNAs in eukaryotes

Name of the eccDNA	Size range	Biological function	References
Mitochondrial DNA	16 kb	Maintaining mitochondria function	134
Double minute	100 kb-3 Mb	Acting as a vehicle for extrachromosomal gene amplification	6, 56
Small polydispersed circular DNA	100 bp-10 kb	Enhancing genomic instability	34
microDNA	100-400 bp	Producing miRNAs	75
Telomeric circle	Integral multiples of 738 bp	Restoring telomere length	135
